# Valorization of Taioba Products and By-Products: Focusing on Starch

**DOI:** 10.3390/foods13152415

**Published:** 2024-07-30

**Authors:** Samanta de Paula de Almeida Duarte, Bárbara E. Teixeira-Costa, Rosely Carvalho do Rosário, Edna Regina Amante, Márlia Barbosa Pires, Orquídea Vasconcelo dos Santos

**Affiliations:** 1 Graduate Program in Food Science and Technology, Institute of Technology, Federal University of Pará, Belém 66075-110, PA, Brazilroselydorosario@gmail.com (R.C.d.R.); e.amante@ufsc.br (E.R.A.);; 2 Department of Nutrition and Dietetics, Fluminense Federal University, Rio de Janeiro 24220-900, RJ, Brazil; betcosta@ufam.edu.br

**Keywords:** unconventional, Xanthosoma sagittifolium, starch sources, ingredient in food, functional properties

## Abstract

Unconventional food plants, popularized in Brazil as PANC, remain underutilized globally. In that sense, this study aims to explore the nutritional and functional properties of taioba (*Xanthosoma sagittifolium*), a plant with edible leaves and tubers, and to investigate its potential for industrial-scale application as a source of starch. A systematic review was carried out and meta-analysis following the PRISMA guidelines was conducted based on a random effects synthesis of multivariable-adjusted relative risks (RRs). The searches were carried out in seven search sources, among which were Web of Science, Elsevier’s Science Direct, Wiley Online Library, Springer Nature, Taylor & Francis, Hindawi, Scielo, ACS—American Chemical Society, and Google Scholar. The systematic review was guided by a systematic review protocol based on the POT strategy (Population, Outcome, and Types of studies), adapted for use in this research. Mendeley was a resource used for organization, to manage references, and to exclude duplicates of studies selected for review. The findings revealed that taioba leaves are abundant in essential nutrients, proteins, vitamins, and minerals. Additionally, the tubers offer rich starch content along with vitamins and minerals like iron, potassium, and calcium, making them an ideal substitute for conventional sources on an industrial scale. This research highlights the significance of studying the functionalities, applicability, and integration of this PANC in our diets, while also emphasizing its capability as a substitute for traditional starch varieties. Moreover, exploiting this plant’s potential adds value to Amazonian resources, reduces import costs, and diversifies resource utilization across multiple industrial sectors.

## 1. Introduction

Unconventional edible plants, referred to as PANC (Plants with Alternative Nutritional Components), encompass a group of nutrient-dense vegetables that are relatively obscure for the general population. Often mistaken for “bushes” or “weeds” [1,2], one noteworthy member of this category is *Xanthosoma sagittifolium*, commonly known as taioba in Brazil or tannia and cocoyam in other regions. Belonging to the Aracacea family, taioba is characterized as a robust leafy vegetable with a towering stature ranging from 1 to 2 m. Its foliage boasts broad, elongated leaves and is accompanied by tuberous roots. Taioba can be found distributed across continents including America, Africa, Asia, and the Pacific Region, showcasing its global adaptability [3,4,5,6].

In Brazil, Taioba is generally cultivated as a family farm, most of the time for personal consumption, using leaves and tubers. But this PANC has become popular in the country and is consumed in restaurants and as an ingredient in bread and flour.

Taioba leaves are known to possess a rich nutrient profile, encompassing essential vitamins such as A and C, along with a plethora of minerals including iron, calcium, potassium, and zinc. Notably, Araújo et al. [7] have advocated for the incorporation of taioba in our diets as a valuable calcium source, potentially aiding in the prevention of diseases such as osteoporosis. Furthermore, the presence of carotenoids and chlorophyll in these compounds endows taioba with distinct anticarcinogenic and antioxidant properties. The versatile nature of taioba extends beyond its nutritional attributes, as demonstrated by Muricy et al. [8], who successfully developed a bread recipe incorporating taioba leaf flour, achieving an exceptional acceptability rate of over 70% in sensory analysis. This noteworthy achievement highlights the significance of introducing novel sources of ingredients in enhancing the functional value of bakery products.

Moreover, the application of taioba in genetic manipulation holds substantial promise, as exemplified by Bansal et al., who employed taioba in in vitro research to facilitate genome multiplication and alteration. The remarkable efficiency of this protocol further emphasizes its potential for enhancing vegetable improvement. The tuberous component of this PANC holds significant scientific interest due to its notable amylaceous content, thereby imparting enhanced energy value to a diet. Notably, the presence of fiber within the tuber serves to regulate glycemia, minimize cholesterol levels, and modulate gut function, further elevating its functional significance. Additionally, it is crucial to emphasize the derived subproducts of this PANC, which offer diverse types of starch reminiscent of corn starch and find applications across various industrial sectors [4,9,10,11,12].

In this regard, scientific research has elucidated the inherent potential and occasional resemblances between unconventional and conventional forms of starch, exemplified by taioba starch. These findings present promising avenues for enhancing feedstock, augmenting the value of food sources, and extending the productive chain. By utilizing subproducts as fundamental raw materials and/or leveraging them for cost reduction purposes while adhering to environmentally friendly practices, this approach furthers the sustainability objectives. From a commercial perspective, the production of pure starch is projected to attain a staggering volume of approximately 156.5 million tons by the year 2025, owing to the varied array of industrially derived products [13,14].

Unconventional starch sources remain largely underexplored both by the general public and the industry, primarily owing to the limited research dedicated to these products. Consequently, traditional starch varieties derived from widely recognized sources such as cassava, corn, potatoes, and wheat dominate the market due to their familiarity to society [15,16]. However, there exists immense potential in investigating and utilizing new starch sources originating from PANC. Expanding knowledge about these alternative products is crucial for their incorporation into the food industry, as it not only enhances nutritional value but also opens avenues for industrial applications. Therefore, it is imperative for the scientific community and society to embrace and expand their understanding of these novel products to unlock their full potential.

Hence, this study aims to explore the nutritional and functional properties of taioba (*Xanthosoma sagittifolium*), a plant with consumable leaves and tubers, and investigate its potential for industrial-scale application as a source of starch.

## 2. Materials and Methods

A systematic review and meta-analysis were conducted following the PRISMA guidelines [17]. Three reviewers independently assessed the methodological quality of the included studies, and there were no disagreements between the reviewers. The primary sources utilized for this review comprised scientific articles sourced from esteemed databases including Web of Science, Elsevier’s Science Direct, Wiley Online Library, Springer Nature, Taylor & Francis, Hindawi, Scielo, ACS—American Chemical Society, and Google Scholar. The searches were carried out using eight sources. The systematic review was guided by a systematic review protocol based on the POT strategy (Population, Outcome and Types of studies) adapted for use in this research. Mendeley was a resource used to organize, manage references, and exclude duplicates of studies selected for review.

For the survey, a comprehensive set of descriptors in both English and Portuguese was employed, encompassing “PANC”, “taioba”, “*Xanthosoma sagittifolium*”, and their alternative nomenclatures, combined with terms such as “Amazonian tuber”, “nutraceuticals”, “fatty acids”, “bioactive”, and “functional foods.” Following the initial search for scientific articles, a subsequent search was conducted by combining the terms “*Xanthosoma sagittifolium*” with “technological properties”, “by-products”, “adding value”, and “different varieties” using the Boolean operator “AND”. The search for scientific articles in the aforementioned databases was not bounded by a specified timeframe nor limited to a particular field of knowledge. In order to achieve the objective, the POT strategy protocol was used to summarize and analyze studies already carried out on the topic in question.

P—Population: Research that addressed the nutritional and functional properties of *Xanthosoma sagittifolium*. The studies included plants with consumable leaves and tubers; excluded were case reports, reviews, descriptive studies, opinion articles, technical articles, editorials, letters to the editor, theses, dissertations, publications from events, books, and book chapters.

O—Outcome: Any type of study indicating benefits to human health from the consumption of *Xanthosoma sagittifolium* or products derived from them, as a source of starch.

T—Type of research: Research that addressed the *Xanthosoma sagittifolium*, indexed in Web of Science, Elsevier’s Science Direct, Wiley Online Library, Springer Nature, Taylor & Francis, Hindawi, Scielo, ACS—American Chemical Society, and Google Scholar, in the form of a scientific article.

### 2.1. Study Selection Process

A calibration exercise was carried out by three reviewers before beginning the study selection process, in which eligibility criteria were reviewed and applied to a 20% sample of retrieved studies to determine inter-rater agreement. The selection process began once the appropriate degree of agreement (Kappa ≥ 0.81) has been reached. Thus, the reviewers chose the studies after reading the titles and abstracts, with no disagreements between the examiners. Subsequently, eligible preliminary studies were obtained and fully evaluated.

### 2.2. Data Extraction

The data extracted from the articles that met the inclusion criteria were analyzed using a Discursive Textual Analysis approach [18] and, after fully reading all studies, the essential data chosen were extracted independently and blinded at the review stage.

## 3. Results

**Figure 1:**
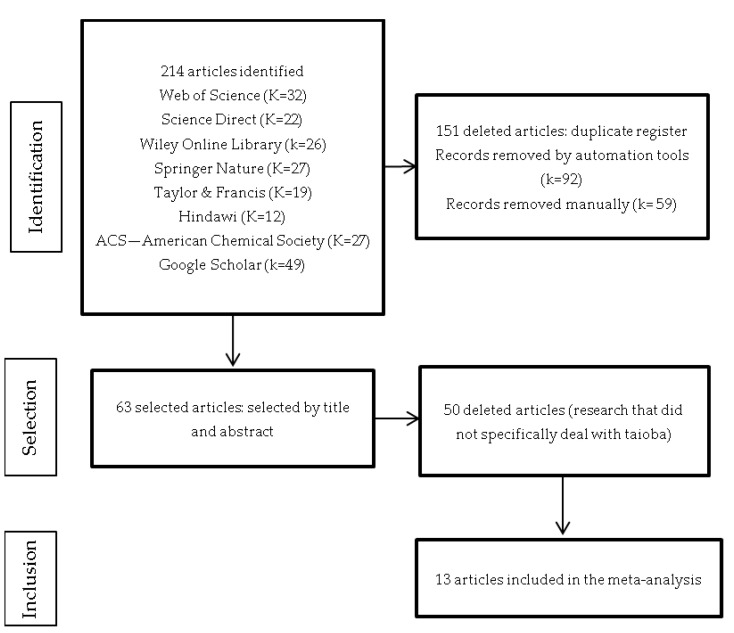
Flowchart of paper selection.

Overall, 214 records were found in the investigated databases. Of these, 151 duplicates were excluded, leaving 63 articles. After reading the titles and abstracts, 50 studies that did not specifically deal with taioba were excluded, leaving 13 for full-text reading. Figure 1 demonstrates the study selection process in detail, following the PRISMA protocol.

Reading the articles made it possible to identify four large groups: taioba leaves, taioba tubers and their products, starch from the taioba tuber, and technological properties of taioba starch (some searches appeared in more than one category). The contents of the research listed will be detailed in Section 4, Section 5, Section 6 and Section 7 of this article and the categories will be analyzed after a brief explanation of the general aspects of taioba.

## 4. General Aspects of the Taioba Plant (*Xanthosoma sagittifolium*)

The Amazonian region boasts remarkable biodiversity, encompassing diverse forms of wildlife and vegetation. Within this realm, the taioba plant (*Xanthosoma sagittifolium*) thrives, earning the designation of an “unconventional food plant” (PANC). This classification, introduced by biologist Valdely Ferreira Kinupp in 2007, pertains to plants that possess edible components and can grow in a spontaneous or cultivated manner. These plants can be of native or exotic origin and are typically geographically limited to specific regions where they exhibit natural development. Notably, PANCs often feature prominently in family farming, cultivated for private consumption [19].

The designation of “unconventional” bestowed upon these plants stems from the dearth of in-depth scientific investigations that substantiate their incorporation into a conventional dietary framework. Nonetheless, PANCs present a viable option in the battle against hunger and in initiatives promoting healthy eating, primarily attributable to their cost-effectiveness and ease of accessibility. Furthermore, these plants assume considerable importance, owing to their remarkable nutritional value, contributing to functions such as enhancing satiety while serving as reservoirs of dietary fibers, vitamins, minerals, and bioactive compounds, which collectively contribute to the prevention of various ailments including cancer, cardiovascular disorders, degenerative diseases, and osteoporosis [2,20,21,22].

**Figure 2:**
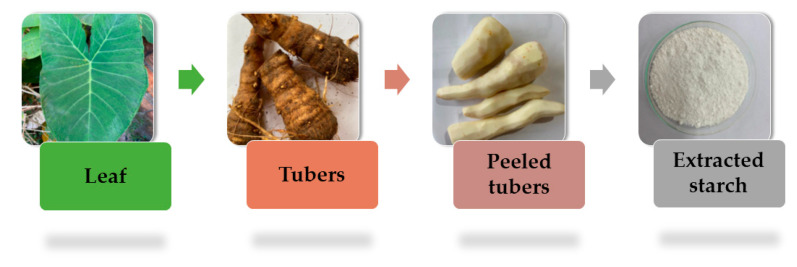
Main processing steps to obtain taioba starch.

Taioba, scientifically known as *Xanthosoma sagittifolium*, is a monocot plant belonging to the Araceae family. It is predominantly distributed across tropical regions in the central part of South America, as well as in select countries in America, Africa, and Asia [3,7,23]. This leafy, perennial, and robust vegetable typically attains a height ranging from 1 to 2 m. Its broad, lengthy leaves exhibit a thick and waxy texture, characterized by prominent veins. The tuber of the plant is found submerged in the soil [24]. Figure 2 provides a visual depiction of the main processing steps to obtain taioba starch.

In Brazil, this unconventional food plant is commonly referred to as taioba, while in other countries it assumes various appellations such as tannia, cocoyam, malanga, and bore, among others. Notably, this species exhibits strong adaptability to elevated temperatures and soils characterized by high levels of humidity. Additionally, it is primarily cultivated for subsistence purposes, given the economically favorable plantation costs and its inherent resilience [25,26].

## 5. Nutritional and Functional Attributes of Taioba Leaves

The nutritional composition of taioba renders it a substantial contributor to dietary value, with both its leaves and tubers being entirely edible. The nutritional composition of taioba leaves is presented in Table 1.

The humidity values were higher than those of caruru (76.17%) and close to ora-pro-nóbis (88.65%) [1]. Protein levels ranged from 2.8 to 4.15 g 100 g^−1^, indicating values lower than caruru (5.58%) and higher than ora-pro-nóbis (2.1%). Lipids are generally low in fruits and vegetables; in this case, they ranged from 0. 20 to 0.62 g 100 g^−1^, values close to those of caruru (0.35%) and ora-pro-nóbis (0.51%) [1]. The fibers present in vegetables (7.38% in caruru, 3.88% in ora-pro-nóbis [1], and up to 11% in taioba) consumed together and with other foods help to supply the daily necessary amount of this component (recommended guidelines established by the World Health Organization—25 g to 30 g per day). The consumption of fiber-rich sources is widely acknowledged to impart numerous benefits for intestinal health, including the promotion of enhanced colonic fermentation, the modulation of the gut microbiota, and the potential to mitigate the occurrence of various diseases such as colon cancer and cardiovascular disorders. Moreover, fiber intake has been associated with a reduction in glycaemia levels and cholesterol [30,31]. Carbohydrate contents ranged from 2.6 to 8.17 g 100 g^−1^, values close to those found in caruru (6.31%) and jambu (4.27%) [1].

When considering the fixed mineral residue content of taioba (as shown in Table 1) and comparing it to other unconventional food plants like lemon vine (*Pereskia aculeata* Miller) [32], the recorded value of 16.7 reveals a comparatively lower concentration.

Such disparities may be attributed to variations in sample preparation, storage conditions, and the methodology employed, as well as environmental factors including climate and soil properties. Nonetheless, collectively, the outcomes presented in Table 1 underscore the noteworthy nutritional potential of this species.

Notably, a comparison of the component values presented in Table 1 reveals distinctive results; these disparities may be attributed to variations in sample preparation, storage conditions, and methodology employed, as well as environmental factors including climate and soil properties, as characteristics of these different plant locations. Nonetheless, collectively, the outcomes presented in Table 1 underscore the noteworthy nutritional potential of this species.

Bioactive compounds are prevalent in vegetables and are synthesized through the plant’s secondary metabolism. These compounds serve essential functions in attracting pollinators and conferring protection against biotic and abiotic factors. Within the human body, these phytochemicals assume significance as they act to neutralize free radicals generated by oxidative stress [33]. Table 2 presents the primary bioactive compounds identified in taioba leaves.

Vitamin C, a non-essential water-soluble micronutrient, plays a vital role in human physiology by performing various functions. These include its antioxidative capacity, the maintenance of the immune system, the promotion of collagen synthesis, the prevention of certain types of cancer, and a reduction in triglyceride and cholesterol levels [30,34]. The studies presented in Table 2 reveal substantial vitamin C content in taioba leaves, particularly in the analysis conducted by Jordan et al. [11] on fresh leaves. These findings are noteworthy as they indicate the potential use of this PANC as a source of vitamin C by pharmaceutical industries. In dry leaves, a sharp drop in vitamin C can be observed due to natural degradation [7,34]. But the values are higher than those of caruru (*Amaranthus deflexus* L.) (28.52 ± 2.46) and major gomes (*Talinum paniculatum* (Jacq.) Gaertn.) (18.4 ± 0.98) leaves [35].

It should be noted that raw plants may contain anti-nutritional factors, which are substances that can hinder nutrient bioavailability by reducing their absorption upon ingestion. In the case of taioba, anti-nutritional factors such as oxalates, trypsin and amylase inhibitors, alkaloids, cyanogens, phytates, and phenols are present. While these compounds may not necessarily be toxic, they can impede the absorption of essential nutrients. Thus, cooking processes are highly effective in reducing the levels of these substances and enhancing the nutritional components’ bioavailability [12,22].

Carotenoids are natural pigments in certain foods that exhibit colors ranging from yellow to red. They serve as antioxidants and as precursors to vitamin A, including β-carotene, α-carotene, and β-cryptoxanthin. This pigment is sensitive to light, temperature and acidity, so values vary depending on the time and method of harvesting and storage. The analysis of the carotenoid data (Table 2) reveals comparable values to other popular unconventional food plants in the Amazon region, such as chicória-do-Pará (*Eryngium foetidum* L., Apiaceae), as observed in the study by Campos et al. [36], yielding an average of 84.3 μg/g, and caruru (*Amaranthus deflexus* L.) (63.76 ± 5.04) [35]. The presence of these pigments is known to promote health by mitigating degenerative diseases like cancer, cardiovascular disease, and macular degeneration, as well as bolstering the immune system [37].

Chlorophyll, the predominant green pigment found in vegetables, garnered the highest value among the works presented in Table 2 in the research conducted by Jordan et al. [11]. This outcome can be attributed to the utilization of raw leaves, an uncommon mode of plant consumption due to the presence of anti-nutritional factors [22]. By comparison, the study by Filho et al. [38] on chicory (*Eryngium foetidum* L.) observed an average result of 30.0 µg/mL, considerably lower than the results reported by the authors listed in Table 2. This disparity in findings may stem from variances in sample preparation, the duration of sun exposure required for chlorophyll production, soil composition, rainfall patterns, leaf morphology and size, and other edaphoclimatic factors [39].

Phenolic compounds are secondary metabolites synthesized by plants, playing crucial roles in pigmentation, insect defense, and pollinator attraction. As portrayed in Table 2, the studies carried out by Jordan et al. [11] and Avelar [34] report higher levels of phenolic compounds. The variations observed in the data presented in Table 2 primarily stem from differences in raw material preparation, as analyzed by Jordan et al. [11] and Avelar [34]. Specifically examining the class of phenolic compounds known as flavonoids, Santos et al. [4] found higher quantities compared to Filho et al. [38] in their analysis of chicory (*Eryngium foetidum* L.) and waterleaf (*Talinum triangulare* Jacq. Willd), yielding 9.12 and 15 mg GAE g^−1^, respectively. These two species are prominent unconventional food plants commonly utilized in the Amazonian diet.

Phenolic compounds play a substantial role in promoting human well-being, as emphasized by Arnoso et al. [40], due to their essential functions in maintaining health. These compounds possess noteworthy antioxidant capacity, contributing to the prevention of diseases such as cancer, Alzheimer’s, and atherosclerosis, aid in reducing cholesterol levels, and exhibit anti-inflammatory effects [41]. The inclusion of vegetables such as taioba in our regular diet is particularly intriguing given their beneficial properties. The studies presented in Table 1 and Table 2 further support this notion, highlighting the potential for diversifying our food choices with nutritionally and functionally rich options. In this context, taioba leaves assume significance not only due to their phenolic compound content but also due to the presence of essential vitamins A and C, as well as an array of minerals including iron, potassium, magnesium, zinc, and calcium, among others [4,7,42]. Table 3 provides an overview of the primary minerals identified in taioba leaves. Minerals play a crucial role in human nutrition, as they are essential nutrients that cannot be produced within the human body and must therefore be obtained through dietary sources. These inorganic substances perform various beneficial functions and are classified as nutrients, categorized into micronutrients and trace elements [42].

The data presented in the studies focusing on taioba leaves (Table 3) provide insights into the potassium, calcium, magnesium, iron, and zinc content. Analysis of the potassium value reveals a variation from 302.86 to 1085.70. The values of plants from Africa are higher than the values detected in Brazil, probably due to the difference in soil. Such values are higher when compared to species prominent in the Brazilian diet, such as chicory (*Eryngium foetidum*) [38] and Caruru (*Amaranthus deflexus* L.) [1]. According to Cukier et al. [45], potassium serves numerous functions in the human body, including for protein and nucleic acid synthesis, cellular growth, and enzyme activation. A deficiency in potassium may result in cardiac arrhythmia, muscle weakness, and glucose intolerance, highlighting the importance of alternative nutrient sources like PANCs.

The variation in magnesium concentrations in studies (Table 3) can be attributed to several factors, such as soil and climate conditions, sample preparation, and storage methods. The values are lower than those presented in caruru and ora-pro-nóbis [1]. Silva et al. [46] and Melo et al. [47] emphasize the significance of magnesium as a nutrient that contributes to numerous functions in the human body, including through antioxidant activity, nervous and muscular maintenance, immune system support, bone integrity, and the stabilization of neuromuscular and cardiovascular membranes. The calcium content in taioba leaves is noteworthy due to its recognized role in bone composition, metabolism, and tissue regeneration, with deficiency leading to issues such as muscle weakness and rickets [48]. These results are remarkable as higher levels of calcium are typically associated with animal-source foods rather than vegetables.

According to Oliveira et al. [49], zinc and iron are relevant as minerals that are required to perform essential biologic activities in the human organism; they act as anti-inflammatories and antioxidants and help to maintain the integrity and functionality of cell membranes, among other things. The predominant sources for these micronutrients are animal-sourced proteins and viscera, among other sources. In the PANC commonly known as lemon vine (*Pereskia aculeata* Mill.), analyzed by Oliveira et al. [50], the authors observed an average of 3.5 mg 100 g^−1^ of zinc, a result that is close to the one observed in taioba leaves [45], confirming how relevant it is to include several PANCs in our regular diet due to their nutritional and functional value, which help in maintaining biological defense systems and the human organic constitution.

## 6. Nutritional and Functional Behavior of the Taioba Tubers and Their Products

The taioba tuber serves as another edible component of this PANC, recognized for being one of the richest sources of carbohydrates, proteins, edible fibers, and vitamins A and C, as well as minerals such as iron, potassium, and calcium, among others. Typically, this tuber is consumed in the form of puree, baked food, or is roasted [3,11]. The tuber of Xanthosoma sagittifolium carries substantial energy value and is fortified with vitamins and minerals, as evidenced by the study conducted by Morais et al. [51], which investigated flour derived from the taioba tuber. The outcomes of this study revealed elevated levels of proteins (12.69%), lipids (1.01%), carbohydrates (74.18%), and minerals (Cu 4.57, Fe 13.00, Mg 6.00, and Zn 28.58% in mg dm^−3^). Based on these findings, the authors recommended utilizing this PANC in the preparation of meat dishes, soups, porridge, and sauces.

Rosida et al. [52] carried out studies with modified taioba tuber flour and the subsequent addition of eggs to formulate non-gluten-based noodles. The first parameter was the proportion of modified taioba tuber flour (90%:10%, 85%:15%, and 80%:20%), and then it was the addition of eggs (5%, 10%, and 15%). The result showed that the best formulation according to chemical, physical, and organoleptic parameters was 85%:15%, with 10% eggs, and this formulation showed both a high dietary fiber content derived from glucomannan (60.14%) and a high protein content (12.75%). In another study, Adedeji and Oluwalana [53] developed a non-alcoholic beverage from taioba tubers, flavored with 0.5% and 1.0% extracts of ginger and alligator pepper, respectively. The results showed that the values of vitamin C content ranged from 1.02 to 1.98 × 10^−4^ mg 100^−1^ g, while vitamin A content ranged from 6.04 to 14.41 μg 100^−1^ g, with ginger-flavored samples having higher vitamin C and A content than the alligator-flavored ones. The minerals evaluated showed decreasing trends with an increase in the concentration of each spice. The sensory results showed that the beverage had good consumer preference, with the 0.5% ginger flavor being the most preferred.

## 7. Starch from the Taioba

Espinosa Solis et al. [31] found that taioba starch possesses a high amylopectin con-tent, rendering it more easily digestible. These authors found higher values of moisture (taioba 15.65%, corn 14.36%), ash (taioba 6.31%, corn 5.69%), protein (taioba 2.79%, corn 0.68%), fat (taioba 1.08, corn 0.11%), and total dietary fiber contents (taioba 1.58, corn 0.36) for taioba starch compared to corn starch, and only the carbohydrate (taioba 74.17%, corn 79.16%) contents were lower. Taioba starch exhibited notable levels of fibers and mineral salts, positioning it as a valuable raw material for extracting resistant starch. Based on the results of their analysis, the authors concluded that taioba starch demonstrated outcomes similar to those of corn starch, making it a potential ingredient for enhancing the nutritional profile of other food products.

Palomino et al. [54] analyzed the proximate composition of taioba starch and identified moisture levels of 10.82%, crude fat at 0.28%, ash at 0.09%, total sugar at 0.05%, starch purity at 99.58%, amylose at 26.17%, and phosphorus at 0.09%. The initial gelatinization temperature of the taioba starch (78.9 °C) was similar to that indicated in the literature, and this value suggests the presence of strong ligations in the interior of the granule. There is also a retrograde trend with a regression of 500 BU. In the micrometry of taioba starch granules, the authors identified a bimodal population with small round, medium truncated ellipsoidal and wide polyhedral collectors (with a media of 5.5 mm) and the presence of a second population of larger size (12–500 mm). They also identified that the distribution of granular size in taioba starch was homogeneous, with 10% for granules between 0.4 and 2.2 mm; 35% in the range of 10.4 to 16.2 mm; and 65% between 2.2 and 10.4 mm. Therefore, this product can be used in the paper industry and in food products where a firm but not clear composition is desired.

Starch, a naturally occurring carbohydrate reserve found in plants, is widely available in the form of minute granules or cells that can vary in size from 1 to 100 μm or larger diameters. The extraction of starch from relevant plants involves a straightforward process consisting of grinding the starch-rich crop and employing wet separation techniques. Pure starch is characterized by its inert nature, exhibiting a white appearance, and being relatively tasteless and odorless. It demonstrates insolubility in cold water and organic solvents like ethanol, ether, and acetone. However, starch is hygroscopic, meaning it has the propensity to absorb water when exposed to normal atmospheric conditions until it reaches an equilibrium state, typically comprising a water content of 10–17% [55].

Starch is a homogeneous polysaccharide comprised of glucose molecules linked by α-1,4 and α-1,6 bonds. It plays a pivotal role in plant storage, being present in intracellular granules within chloroplasts as assimilation starch and in leucoplasts or amyloplasts as reserve starch. During photosynthesis, glucose is accumulated and deposited in various plant parts such as leaves, seeds, fruits, and tubers [55]. Notable examples of starch-containing foods include cereals like rice, corn, and wheat, tubers and roots like potato and cassava, and legumes like beans, lentils, peas, and chickpeas, among others [56,57].

The structure of starch encompasses two polysaccharides, namely amylose and amylopectin, whose qualities and functionalities are influenced by the proportion, chain length, and organization of their constituent molecules. These characteristics differ depending on the botanical source of each starch type [58,59]. Amylose is a linear polymer in which glucose molecules are connected by glycosidic bonds in an α-(1→4) arrangement. It consists of approximately 200 to 10,000 glucose molecules and possesses a molecular weight of approximately 1 × 10^6^ g mol^−1^ [60]. On the other hand, amylopectin adopts a highly branched structure with α-(1→6) connections. It is regarded as one of the largest molecules found in nature, with a molecular weight ranging from 1 × 10^7^ to 1 × 10^9^ g mol^−1^ and containing 5000 to 50,000 glucose molecules [61,62].

The morphology of starch granules, including their shapes (round, oval, and polyhedral), sizes (ranging from 2 to 100 μm), and distribution patterns (such as unimodal, bimodal, and trimodal), are determined by the botanical characteristics of the plant species from which the starch is extracted [63,64]. Crystallinity, another key aspect of starch classification, is determined by the arrangement of granule rings, which exhibit alternating crystalline and amorphous regions. Starch can be classified as either crystalline or semi-crystalline, with three polymorphs (A, B, and C) identified. Type A represents a highly condensed and crystalline amylopectin structure commonly found in cereals. Type B is characterized by hexagonal arrangements of basic chain units and is typically found in starch derived from tubers. Type C serves as an intermediate polymorph between types A and B and is commonly found in roots and seeds [65].

Taioba starch granules exhibit an ovoid and circular morphology, with dimensions ranging from 4.23 µm to 9.93 µm, and possess smooth surfaces. In a study by Ramos et al. [12], the dimensions of taioba starch were reported to range approximately from 3.6 µm to 14 µm, displaying circular shapes. Comparatively, taioba starch granules are similar in size to maize (*Zea mays* L.) starch granules, which were measured to be approximately 10 µm in a study conducted by Cesar et al. [66]. According to the authors, this similarity in size is advantageous for industrial applications, as small-sized starch particles, around 2 µm, can serve as a fat substitute due to their particle size resemblance.

The circular shape of taioba starch shares similarities with starch granules obtained from edible banana, as observed in a study by Takeiti et al. [67]. Similarly, bamboo starch (*Dendrocalamus asper*) was found to possess smooth surfaces and circular granules [68]. Peach palm fruit (*Bactris gasipaes* Kunth) starch granules exhibit an oval and rounded shape, with dimensions ranging from 2 µm to 10 µm, which closely aligns with taioba starch characteristics [16]. Barros et al. [69] also documented the spheric-shaped granules and smooth surface of Guinea arrowroot (*Goeppertia allouia*) starch.

The size and shape of starch granules play a significant role in determining their technological properties, such as gelatinization, swelling, and solubility. Smaller and more diverse granule sizes facilitate better distinguishability by amylases, resulting in enhanced digestibility [67,70]. These factors highlight the importance of understanding the size and shape characteristics of starch granules, as they have implications for various applications in the food industry.

## 8. Technological Properties of Taioba Starch

Brazil boasts a rich biodiversity of flora and fauna, particularly in terms of dietary properties. However, many of these plant species remain underutilized economically and have yet to penetrate the market at a significant scale. Consequently, there is a dearth of information regarding their nutritional, functional, and technological potential [15]. In this context, non-conventional starch sources derived from plants with high nutritional value are emerging. These plants, despite being relatively unexplored by the food industry, hold significant potential. Moreover, the pursuit for nutritious and appealing food options has led to an increasing interest in starch within the food industries. This interest is driven not only by the physical and chemical characteristics of starch but also by its nutritional appeal. One important component contributing to this appeal is the presence of resistant starch, a fraction of starch that shares similarities with dietary fiber and can offer valuable benefits in various technological processes within the food industry [63,64,65].

Resistant starch constitutes a minor fraction of starch that resists digestion in the small intestine and instead reaches the colon, where it serves as a substrate for the bacterial flora [71,72]. The numerous advantages of resistant starch encompass improved digestion and prebiotic effects, as well as anti-inflammatory and anticarcinogenic properties, among others [73,74,75]. Furthermore, due to its functional properties, resistant starch finds application in the food industry for the fortification of dietary fibers, the enhancement of texture and sensory qualities, and even in the production of biodegradable films [76].

**Figure 3:**
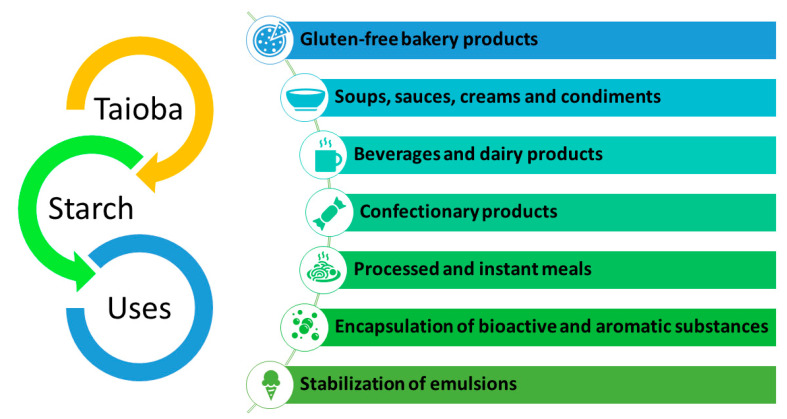
Potential food-related applications of taioba starch.

Borba et al. [63] and Castro et al. [77] categorize starch into native and modified forms based on its applications in industries such as paper, children’s food, and chemical yeast production. Native starch refers to unmodified starch, while modified starch encompasses the chemically, physically, or enzymatically modified variants utilized in the production of sweeteners, biodegradable plastics, dextrin, and other products. Fermented starch, in turn, primarily serves as a precursor for sour starch production. These classifications highlight the diverse sources of starch utilized by industrial sectors, which undergo processing across various industries. The natural properties of starch render it highly valuable for application across different industrial sectors [78]. Figure 3 presents several noteworthy examples illustrating the industrial uses of starch.

Research dedicated to exploring various sources of starch plays a vital role in enhancing the utilization of raw materials within significant industrial sectors. These endeavors seek to leverage starch as a fundamental component in both the initial stages of the production chain and the final stages, enabling the manufacture of cardboard, edible films, biodegradable packaging, and other related products. By doing so, the aim is to mitigate the adverse environmental effects associated with the use of non-degradable packaging materials [79,80,81]. Table 4 showcases several examples of such amylaceous plants that not only contribute to the fight against hunger but also represent easily obtainable food sources. Among these plants are arrowroot (*Maranta arundinaceae* L.), guinea arrowroot (*Goeppertia allouia*), bamboo (*Dendrocalamus asper*), air potato (*Dioscorea bulbifera*), babassu palm (*Orbignya speciosa*), peach palm (*Bactris gasipaes* Kunth), taioba (*Xanthosoma sagittifolium*), and uvaia (*Eugenia pyriformis*) [12,16,68,82,83]. These unexplored plant species hold great potential for addressing food security concerns and can contribute to the diversification of the food supply.

In their investigation, Pires and colleagues [16] employed two distinct methods, namely the aqueous method and the alkaline method, to extract starch from the peach palm fruit (*Bactris gasipaes* Kunth). The results of the aqueous extraction on the mesocarp exhibited a relatively low amylose content (3.46 ± 0.11 g 100 g^−1^), while the amylopectin content was significantly higher (96.54 ± 0.57 g 100 g^−1^). This classification as a waxy starch, has garnered substantial attention from the industrial realm. Consequently, there is substantial support for utilizing peach palm fruit starch in bakery products. Furthermore, the alkaline extraction method yielded superior starch performance compared to the aqueous method, underscoring the recommendation for employing both methods in the production of pharmaceutical and chemical products.

In the study conducted by Felisberto et al. [68], the findings demonstrated the presence of a thermally stable starch with high gelatinization potential. As a result, the authors emphasize the use of bamboo starch (*Dendrocalamus asper*) as a potential alternative to conventional feedstocks like corn and rice. The research by Felisberto et al. [83] has highlighted the significant role of bamboo in various sectors, including functional food, pharmaceutical industries, and biofuel production. According to Silva et al. [70], air potatoes (*Dioscorea bulbifera*) serve as a valuable source of minerals, proteins, and carbohydrates, as well as moisture, fibers, and lipids. Notably, the exceptional technological properties of starch enable its efficient utilization in the production of bakery products, thus facilitating the promotion of air potato cultivation in the Amazon region [85].

Farias et al. [86] and Rodrigues et al. [86] have reported the presence of phytochemical compounds in uvaia (*Eugenia pyriformis* Cambess), which contribute to its nutritional composition and confer antioxidant, antimicrobial, and anti-inflammatory activities. These qualities make uvaia a promising ingredient for utilization in pharmaceutical, cosmetic, and food products, thereby fostering industrial advancements through innovative manufacturing processes. Carvalho et al. [82] and Barros et al. [68] have also discovered the considerable potential of Guinea arrowroot (*Goeppertia allouia*) in the food and pharmaceutical industries, underscoring the significance of exploring new sources of feedstock from the Amazon region.

The studies conducted by Moura et al. [83] and Maniglia et al. [88] have demonstrated the advantageous effects of incorporating babassu palm starch (*Orbignya speciosa*) into the regular diet, enhancing its status as a functional food. This substantiates its significance in enhancing the nutritional value of various products. Additionally, babassu palm starch exhibits promising potential in the realm of packaging as it can be utilized in the production of biodegradable films. Furthermore, the research carried out by Tresina et al. [89] and Ramos et al. [12] focuses on the exploration of taioba starch (*Xanthosoma sagittifolium*) and highlights its nutritional importance. Moreover, the analysis of taioba starch reveals its morphological characteristics, resembling those of amylopectin, classifying it as a waxy starch. This unique feature enables its applicability across diverse industrial sectors.

## 9. Conclusions: Unlocking the Potential of Taioba

The literature on taioba (Xanthosoma sagittifolium), focusing on its edible leaves and tubers, as well as its subproducts such as starch, has allowed us to assess the current state of knowledge regarding this plant. Despite its potential, taioba has been minimally utilized in regional culinary practices and experimental research efforts. This study significantly contributes to the expansion of the scientific, nutritional, and functional knowledge of taioba. It presented various studies on different aspects of taioba, including its parts, functionalities, and technological applications. This research opens up opportunities for the development of new products using taioba as a base component or ingredient. The review of recent research, including this present study, provides a foundation for further in-depth investigations into taioba. Analyzing the nutritional and functional potential of taioba components separately or as a whole is crucial. Additionally, exploring its potential for industrial use can help diversify feedstocks, add value to the production chain, and contribute to environmental preservation by reducing waste.

Potential areas of research include investigating new phytochemical increments, protein resources, amylaceous components, and gelatin-based products derived from taioba. These products can be applied in active packaging, as antimicrobial agents, and in the production of nutraceutical products, fiber-based probiotics, and nanomaterials for hygienic food management. There is a growing demand for studies that utilize sensitive technologies to track bioactive components through various extraction and isolation techniques. This demand aligns with the principles of green chemistry, which prioritize the use of techniques and reagents derived from natural sources. These studies have important implications for pharmaceutical, dermo-cosmetic, and dietary components, with a particular emphasis on their functional properties in the context of nutraceutical products. Consumers have shown an increasing interest in these functional products, thus highlighting the importance of conducting further research in this area.

In the case of taioba, a highly underutilized PANC, it is primarily employed as a culinary ingredient in traditional diets, with a focus on its leaves and stems. This limited use overlooks its rhizome, which contains a high concentration of starch. By exploring the potential of the taioba rhizome and adopting green practices in its extraction, industries and the general public can benefit from reduced food production costs. Incorporating new feedstocks into starch-based product development across various sectors can foster economic efficiency and sustainability. In conclusion, the presented research provides valuable insights into the untapped potential of taioba. It highlights the need for further studies to fully understand the nutritional and functional properties of taioba and explores the possibilities of utilizing its various components in the development of new products. The application of sensitive technologies and green chemistry principles can enhance the extraction and isolation of bioactive components from taioba, opening up opportunities for pharmaceutical, dermo-cosmetic, and dietary research.

Utilizing taioba as a base component or ingredient in food products can contribute to the diversification of feedstocks and the creation of value-added products. This not only benefits the industries involved but also supports environmental preservation by reducing waste. Additionally, the demand for functional and nutraceutical products by consumers creates a market opportunity for taioba-based products. By considering the underutilized aspects of taioba, such as its rhizome with its high starch content, researchers can contribute to the development of new starch-based products. This can potentially reduce the cost of food production and promote the implementation of green practices. Hence, further research on taioba is essential to maximize its potential as a PANC. By exploring its nutritional and functional properties, adopting green chemistry principles, and considering its various components for new product development, we can unlock the opportunities taioba offers in the fields of food, pharmaceuticals, and cosmetics.

## Figures and Tables

**Table 1:** Nutritional composition of taioba leaves.

Nutritional Composition (g 100 g^−1^)	Souza et al. [27]			Moura et al. [28]	Siqueira et al. [29]
	Raw	Cooked			
Moisture *	82.00	92.00	86.58	90.77	85.00 ± 0.11
Minerals	ND **	ND **	1.74	1.57	3.45 ± 0.71
Lipids	0.20	0.20	0.62	0.51	0.75 ± 0.05
Proteins	3.00	2.80	3.05	3.08	4.15 ± 0.02
Carbohydrates	2.60	2.90	4.12	4.07	8.17 ± 0.15
Fibers	11.00	1.10	3.89	ND **	ND **
Calorific value (kcal 100 g^−1^)	24.20	24.60	34.26	ND **	ND **

* Values expressed on a wet basis; ** ND: Not determined.

**Table 2:** Some bioactive compounds found in taioba leaves.

Bioactive Compounds	Araújo et al. [7]	Jordan et al. [11]	Avelar [34]
Ascorbic acid (mg 100 g^−1^)	87 ± 0.79	561.60 ± 0.73	58.30 ± 0.82
Carotenoids (mg g^−1^)	83.19 ± 0.54	ND *	36.05
Total chlorophyll (mg 100 g^−1^)	8.94 ± 0.02	383.22 ± 0.01	272.81
Phenolic compounds (mg GAE g^−1^)	5.33 ± 0.18	83.20 ± 0.82	2.61
Flavonoids (mg 100 g^−1^)	ND *	ND *	55.00

ND *: Not determined. Values are expressed as mean ± standard deviation.

**Table 3:** Some minerals found in taioba leaves.

Micronutrients (mg 100 g^−1^)	Botrel et al. [1]	Ndabikunze [43]	Wada [44]
Sodium	1.05	8.39 ± 0.22	29.22 ± 1.44
Potassium	302.86	760.21 ± 29.00	1085.70 ± 32.10
Magnesium	0.32	0.783 ± 0.05	2.48 ± 0.19
Calcium	77.63	76.66 ± 11.70	56.57 ± 1.50
Manganese	23.82	69.53 ± 4.90	78.77 ± 0.67
Iron	1.17	3.28 ± 0.19	8.20 ± 0.60
Zinc	0.21	1.35 ± 0.13	3.07 ± 0.10
Copper	0.10	0.43 ± 0.45	1.04 ± 0.08
Phosphor	ND *	277.76 ± 9.015	120.93 ± 2.07

ND *: Not determined.

**Table 4:** Some sources of non-conventional starches and their nutritional content.

Source	Moisture (g 100 g^−1^)	Lipids (g 100 g^−1^)	Minerals (g 100 g^−1^)	Proteins (g 100 g^−1^)	Apparent Amylose (g 100 g^−1^)	Starch Yield (%)	Reference
*Bactris gasipaes*	10.63 ± 0.20–12.69 ± 0.21	0.47 ± 0.03–1.04 ± 0.06	0.11 ± 0.03–0.15 ± 0.01	0.35 ± 0.04–0.49 ± 0.02	1.07 ± 0.06–3.99 ± 0.01	28.25 ± 3.19–61.70 ± 1.91	[16]
*Dendrocalamus asper*	5.88 ± 0.4–6.88 ± 0.65	0.21 ± 0.04–0.46 ± 0.08	0.90 ± 0.01–1.13 ± 0.02	1.76 ± 0.02–2.17 ± 0.03	12.03 ± 0–29.71 ± 0	11.06 ± 1.90–15.19 ± 1.47	[68]
	3.73 ± 0.09–4.65 ± 0.13	0.19 ± 0.12–0.22 ± 0.03	0.36 ± 0.01–0.72 ± 0.02	0.77 ± 0.02–1.04 ± 0.03	ND *	1.01 ± 0.17–3.94 ± 0.07	[84]
*Dioscorea alata* and *D. altissima.*	9.67 ± 0.09–13.46 ± 0.01	0.57 ± 0.03–0.83 ± 0.04	0.06 ± 0.00–0.11 ± 0.02	0.77 ± 0.00–0.80 ± 0.01	17.91 ± 0.01–19.15 ± 0.01	7.76–8.57	[70]
*Dioscorea bulbifera*	10.43 ± 0.02–12.06 ± 0.01	0.85 ± 0.13–1.63 ± 0.58	2.14 ± 0.03–2.70 ± 0.03	13.83 ± 0.29–16.53 ± 0.26	ND *	57.77 ± 3.73–75.70 ± 2.92	[85]
*Eugenia pyriformis*	91.75 ± 0.13	2.95 ± 0.17	3.63 ± 0.14	17.85 ± 0.23	ND *	ND *	[86]
	76.0 ± 1.0	0.35 ± 0.006	0.31 ± 0.015	2.66 ± 0.006	ND *	ND *	[87]
*Goeppertia allouia*	7.02 ± 0.38	0.028 ± 0.01	0.03 ± 0.02	0.026 ± 0.4	ND *	13.145 ± 0.06	[82]
	8.45 ± 0.04	0.39 ± 0.01	0.15 ± 0.02	2.4 ± 0.1	38.6 ± 1.6	ND *	[70]
*Orbignya speciosa*	6.35 ± 0.14	0.72 ± 0.03	0.31 ± 0.01	1.02 ± 0.06	36.51 ± 1.02	ND *	[83]
	15.1 ± 1.6	1.8 ± 0.4	1.1 ± 0.1	1.4 ± 0.1	36.6 ± 0.5	ND *	[88]
*Xanthosoma* *sagittifolium*	72.16 ± 0.76	6.32 ± 0.12	4.68 ± 0.08	9.68 ± 0.05	ND *	ND *	[89]
	77.1 ± 2.82	0.2 ± 0.06	1.1 ± 0.09	4.4 ± 0.42	ND *	ND *	[12]

ND *: Not determined.

## Data Availability

The original contributions presented in the study are included in the article, further inquiries can be directed to the corresponding author.
